# An ADAR1-dependent RNA editing event in the cyclin-dependent kinase CDK13 promotes thyroid cancer hallmarks

**DOI:** 10.1186/s12943-021-01401-y

**Published:** 2021-09-08

**Authors:** Julia Ramírez-Moya, Christos Miliotis, Allison R. Baker, Richard I. Gregory, Frank J. Slack, Pilar Santisteban

**Affiliations:** 1 Instituto, de Investigaciones Biomédicas “Alberto Sols”; Consejo Superior de Investigaciones Científicas (CSIC), Universidad Autónoma de Madrid (UAM), Madrid, Spain; 2 Department of Pathology, Harvard Medical School Initiative for RNA Medicine, Beth Israel Deaconess Medical Center, Harvard Medical School, Boston, MA USA; 3 Stem Cell Program, Division of Hematology/Oncology, Boston Children’s Hospital, Departments of Biological Chemistry and Molecular Pharmacology, and Pediatrics, Harvard Medical School, Harvard Medical School Initiative for RNA Medicine, Boston, MA USA; 4 Centro de Investigación Biomédica en Red de Cáncer (CIBERONC) Instituto de Salud Carlos III (ISCIII), Madrid, Spain

**Keywords:** ADAR1, A-to-I editing, Cancer, CDK13, Splicing

## Abstract

**Background:**

Adenosine deaminases acting on RNA (ADARs) modify many cellular RNAs by catalyzing the conversion of adenosine to inosine (A-to-I), and their deregulation is associated with several cancers. We recently showed that A-to-I editing is elevated in thyroid tumors and that ADAR1 is functionally important for thyroid cancer cell progression. The downstream effectors regulated or edited by ADAR1 and the significance of ADAR1 deregulation in thyroid cancer remain, however, poorly defined.

**Methods:**

We performed whole transcriptome sequencing to determine the consequences of ADAR1 deregulation for global gene expression, RNA splicing and editing. The effects of gene silencing or RNA editing were investigated by analyzing cell viability, proliferation, invasion and subnuclear localization, and by protein and gene expression analysis.

**Results:**

We report an oncogenic function for *CDK13* in thyroid cancer and identify a new ADAR1-dependent RNA editing event that occurs in the coding region of its transcript. *CDK13* was significantly over-edited (c.308A > G) in tumor samples and functional analysis revealed that this editing event promoted cancer cell hallmarks. Finally, we show that *CDK13* editing increases the nucleolar abundance of the protein, and that this event might explain, at least partly, the global change in splicing produced by ADAR1 deregulation.

**Conclusions:**

Overall, our data support A-to-I editing as an important pathway in cancer progression and highlight novel mechanisms that might be used therapeutically in thyroid and other cancers.

**Supplementary Information:**

The online version contains supplementary material available at 10.1186/s12943-021-01401-y.

## Introduction

Adenosine deaminases acting on RNA (ADARs) are involved in the most common type of RNA editing in mammals, A-to-I RNA editing [1]. Acting specifically on double-stranded RNAs, ADARs catalyze the post-transcriptional conversion of adenosine residues to inosines, which are recognized by the cellular machinery as guanosines. Accordingly, A-to-I editing can create, delete or alter the specificity of a codon or a splice site, alter RNA structures, or modify regulatory RNAs [1]. Importantly, when this occurs in coding regions it can lead to the generation of new proteins with novel functions [2]. As A-to-I RNA editing overlaps temporally and spatially with pre-mRNA splicing, it is likely that extensive crosstalk exists between the two processes. Indeed, A-to-I RNA editing has been shown to globally affect alternative splicing [3, 4]; however, only a limited number of editing sites directly affect splice sites, and it is highly possible that additional mechanisms exist to regulate splicing [3, 4]. Although poorly understood, RNA editing events within transcripts of genes related to splicing regulation might explain the global changes in splicing induced by the ADARs. ADAR1, which is expressed through two isoforms (p110 and p150) [2], is the most abundant member of the ADAR family of enzymes and plays important roles in both physiological and pathological processes.

RNA editing drives molecular diversity, offering an organism the potential for greater complexity. ADAR editing is essential for survival in mammals, but its deregulation is also associated with cancer initiation and progression [5, 6]. Recent studies, especially those by The Cancer Genome Atlas (TCGA) project, have established that A-to-I editing levels and the enzymes mediating this modification are significantly altered in cancer [7, 8]. Most tumor types have elevated levels of A-to-I editing and ADAR1 expression, and this latter finding has been associated with a reduction in patient survival in glioma, papillary thyroid and uterine corpus endometrial carcinomas [9]. The available data also suggest that edited RNAs might serve as novel candidates for therapeutic and diagnostic purposes [8]. In this context, we recently demonstrated the importance of A-to-I editing in thyroid cancer [10], where ADAR1-dependent activity is markedly higher in tumors than in normal thyroid tissue [7, 8]. Thyroid cancer is the most common endocrine malignancy [11], and its incidence has increased significantly in the US and in other countries over the last few decades [12–14]. Although it generally has a good outcome, the rate of disease recurrence is high, which is associated with increased incurability and with patient morbidity and mortality [15]. Indeed, some patients develop aggressive forms of the disease that are untreatable, and the molecular foundations remain poorly understood [15]. Accordingly, a better understanding of thyroid cancer is essential to develop new strategies and provide new therapeutics for treatment. Thyroid cancer encompasses several histological types and subtypes with different cellular origins, characteristics and prognoses. Most tumors originate from follicular cells and can be classified as well-differentiated carcinomas, including papillary (PTC) and follicular (FTC) thyroid carcinoma, whereas others are classified as poorly differentiated (PDTC) and undifferentiated or anaplastic (ATC) thyroid carcinomas [16, 17]. The latter, less-differentiated, thyroid cancers (PDTC and ATC) can develop de novo, although many arise through the process of stepwise differentiation of PTC and FTC [17].

We previously demonstrated a functional role for ADAR1 and RNA editing in thyroid cancer tumorigenesis following *ADAR1* gene silencing and pharmacological inhibition of ADAR1 editase activity. We also found that some microRNAs, such as miR-200b, are new targets for ADAR1 in thyroid cancer [10]. Still, several issues remain unresolved regarding how RNA editing affects thyroid cancer. In the present study, we used bioinformatic approaches and high throughput RNA-sequencing (RNA-seq) of *ADAR1* knockdown cancer cells to globally examine how ADAR1 and its A-to-I RNA editing activity influences gene expression and mRNA splicing. This analysis allowed us to identify novel editing sites for ADAR1 in the transcriptome and uncover a new ADAR1-dependent RNA editing event that occurs in *CDK13*, which encodes a cyclin-dependent kinase implicated in the regulation of transcription [18] and RNA splicing [19, 20]. We found that the nucleotide modification from this editing event is overrepresented in thyroid tumors and has functional consequences in thyroid cancer cells. Overall, our data point to an oncogenic role for *CDK13* in thyroid cancer, as the ADAR1-dependent editing of *CDK13* provides an advantage for cancer progression and may explain the global change in splicing pattern observed upon *ADAR1* knockdown.

## Materials and methods

### Patients

Samples of paired PTC tumors and contralateral normal thyroid tissue from patients (*n* = 6) were obtained from the Biobank of the La Paz University Hospital (Madrid, Spain). The main clinical characteristics of the patients have been described [10]. Informed consent was obtained from all the patients following the protocols approved by the hospital ethics committee.

### Cell culture

Cal62 and TPC1 tumor thyroid cell lines were grown in Dulbecco’s modified Eagle’s medium (DMEM) supplemented with 10% fetal bovine serum (FBS). The Cal62 cell line was obtained from Leibniz-Institut DSMZ-German Collection of Microorganisms and Cell Cultures and the TPC1 cell line was provided by Dr. A.P. Dackiw (Johns Hopkins University, Baltimore). Cells were tested for mycoplasma contamination and authenticated every 6 months by short tandem repeat profiling using the Applied Biosystems Identifier kit in the Genomic Facility at the Institute of Biomedical Research (IIBm, Madrid, Spain).

### siRNAs, plasmids and transfections

The following siRNAs were purchased from Thermo Fisher Scientific Inc. (Waltham, MA): siControl (Silencer Select Negative Control #1 4,390,843), siADAR1 #1 (s1007), siADAR1 #2 (119,581), siCDK13 #1 (s16397), siCDK13 #2 (s16398), siCDK13 #3 (s16399). All siRNAs were transfected using Lipofectamine RNAimax (Thermo Fisher Scientific). Lentiviral plasmids expressing the wild-type (WT) CDK13, the c.308A > G edited (edit) form or the empty plasmid were designed and constructed by VectorBuilder Inc. (Chicago, IL). Puromycin was used for selection.

### RNA sequencing

Total RNA was isolated from transfected cells using the mirVana Isolation Kit (Ambion, Austin, TX) and was processed at the Dana Farber Molecular Biology Core Facility (two biological independent replicates). RNA quality was confirmed on the Agilent Bioanalyzer (Santa Clara, CA). Library preparation was performed using the KAPA mRNA HyperPrep Kit for poly(A +) (Roche; Basel, Switzerland). RNA-seq analysis was performed on the Illumina NextSeq 500 platform (San Diego, CA) using 75-bp paired-end reads. The data have been deposited in the NCBI Gene Expression Omnibus [21] and are accessible through the GEO Series accession number GSE165282 (https://www.ncbi.nlm.nih.gov/geo/query/acc.cgi?acc=GSE165282).

### Differential expression analysis

Differential expression comparisons were performed for siControl *versus* siADAR#1 and siADAR#2. All samples were processed using an RNA-seq pipeline implemented in the bcbio-nextgen project (https://bcbio-nextgen.readthedocs.org). Raw reads were examined for quality issues using FastQC (http://www.bioinformatics.babraham.ac.uk/projects/fastqc/) to ensure that library generation and sequencing were suitable for further analysis. Adapter sequences and other contaminant sequences were trimmed from reads using Atropos [22]. Counts of reads aligning to known genes were generated by featureCounts [23]. In parallel, Transcripts Per Million (TPM) measurements per isoform were generated by quasialignment using the Salmon tool [24]. Normalization at the gene level was called with DESeq2 [23, 25], with preference to use counts per gene estimated from the Salmon quasialignments by Tximport [23, 25, 26]. The DEGreport Bioconductor package was used for quality control and clustering analysis (https://doi.org/10.18129/B9.bioc.DEGreport). DESeq2 was used for differential expression analysis.

### Variant calling analysis

BAM files were processed with GATK [27] following the best-practices for RNA-seq variant calling, to compile a list of nucleotide variants in each sample. In addition, we added an additional filter to remove calls within 10 bases of a junction on either side. Variants were annotated with the SnpEff tool [28]. For differential allele frequency analysis, we removed all annotated single nucleotide polymorphisms (SNPs), and fitted a linear model to the allele frequency values from the two groups: siADAR1 #1/2 and siControl. We employed the Benjamin-Horchberg method for p-value correction to deal with multiple testing.

### Splicing analysis

Differential splicing analysis was performed using Multivariate Analysis of Transcript Splicing (rMATS) (http://rnaseq-mats.sourceforge.net/) with default parameters. The RNA-seq reads were mapped to the human genome assembly GRCh38 using the STAR aligner. rMATS evaluates splicing per sample in two ways: by counting only the number of reads that map to the splice junctions (JC analysis), and by also counting the reads that map within the alternately spliced target region (JCEC analysis). The JCEC output was used for further analysis. Differential splice comparisons were performed for both siControl *versus* siADAR#1 and siControl *versus* siADAR#2. Inclusion and exclusion junction reads were averaged from replicates and used to calculate the Inclusion Level Difference (PSI score) for each splice site. Hits were filtered by removing sites with < 15 reads total in either sample average (siControl or siADAR1) and by using a false discovery rate (FDR) cut-off of < 0.05.

### Functional annotation of candidate genes

The genes obtained after the RNA-seq analysis were processed by The Database for Annotation, Visualization and Integrated Discovery (DAVID, http://david.abcc.ncifcrf.gov) for functional annotation.

### RNA quantification

For gene expression analysis, total RNA was isolated with Trizol Reagent (Invitrogen). Template cDNA synthesis was performed using the NZY M-MuLV First-Strand cDNA Synthesis Kit (Nzytech, Lisbon, Portugal). Quantitative reverse transcription-PCR (qRT-PCR) was performed with the KAPA SYBR Fast Universal Kit from Sigma-Aldrich. (Madrid, Spain) All primers were purchased from Sigma-Aldrich and are described in Table S1.

### Verification of RNA editing sites

The RNA editing site c.308A > G was verified using RT-PCR with the following PCR primers: forward primer: 5’-CTCTGGAGGTCAAGCGGC-3’ and reverse primer: 5’-GACTGGGAGCTCACATCCTC-3’. PCR products were evaluated by Sanger sequencing and editing levels were calculated with INDIGO (https://www.gear-genomics.com/indigo/).

### Protein extraction and western blotting

Cells were lysed and proteins extracted with RIPA buffer [29]. Protein concentration was measured using the Bradford method (Bio-Rad Laboratories, Hercules, CA). Samples were separated by sodium dodecyl sulfate–polyacrylamide gel electrophoresis (SDS-PAGE) and transferred to nitrocellulose membranes (Bio-Rad). Immunoreactive proteins were visualized by enhanced chemiluminescence (GE Healthcare Bioscience, Pittsburgh, PA).

### Immunofluorescence

Cells were seeded on coverslips for 48 h, washed with phosphate-buffered saline (PBS), fixed in 4% paraformaldehyde for 15 min, permeabilized with PBS containing 0.1% NP40 for 10 min, washed again and then blocked at room temperature with PBS-5% Tween containing 3% bovine serum albumin and 1:100 normal goat serum for 30 min. Coverslips were incubated with a 1:100 dilution of primary antibodies in blocking solution overnight at 4 °C, washed three times in PBS-5% Tween for 5 min and incubated for 1 h at room temperature with the secondary antibodies (Jackson Immunoresearch, West Grove, PA). After rinsing three times with PBS-5% Tween, with the last wash containing 1:5,000 4,6-diamidino-2-phenylindole (DAPI), cells were finally mounted on Prolong antifade reagent (Invitrogen, Carlsbad, CA). Cells were observed using a confocal microscope with × 63 oil immersion objective; × 2 zoom was used for Cal62 cells. Colocalization was analyzed using the Fiji-Coloc2 plugin.

### Antibodies

The following antibodies were obtained from Santa Cruz Biotechnology Inc. (Santa Cruz, CA): SP1 (sc-17824), tubulin (sc-5286) and vinculin (sc-73614). CDK13 (ab251955) and Fibrillarin (ab4566) antibodies were purchased from Abcam (Cambridge, UK). The GAPDH (MAB374) antibody was from EMD Millipore Corp. (Billerica, MA) and the hemagglutinin (HA) antibody (C29F4) was from Cell Signaling Technologies, (Danvers, MA).

### Proliferation and cell viability assays

Cell proliferation was measured by DNA synthesis using bromodeoxyuridine (BrdU) labeling with an assay from Sigma-Aldrich (#11,669,915,001). Briefly, cells were seeded in 96-well plates, pulse-labeled for 5 h with 10 μM BrdU, and BrdU incorporation was measured in a luminometer (Promega, Madison, WI) [30]. Cell viability was determined using an XTT metabolic assay (Canvax Biotech, Córdoba, Spain) and crystal violet staining. For XTT analysis, cells were seeded in 96-well plates and dye reduction was recorded on a spectrophotometer after 72 h. For crystal violet staining, Cal62 and TPC1 cells were seeded in each well of a 6-well plate. Individual wells were fixed in 4% paraformaldehyde after 7–10 days and stained with crystal violet. After extensive washing and drying, the staining reagent was resolubilized in 1% acetic acid and quantified at 590 nm as an indirect measure of cell number.

### Invasion assay

Cell invasion was assayed in Transwell cell culture chamber filters coated on the upper side with Matrigel (Corning BioCoat, Corning, NY), as described [29, 30]. Briefly, 45,000 Cal62 cells or 50,000 TPC1 cells in DMEM containing 0.2% FBS were seeded in the upper chamber and 20% FBS was added to the bottom chamber as a chemoattractant. Cells were allowed to invade for 22 h. For the analysis of cell invasion using the CDK13-WT and -EDIT constructs, a total of 35,000 Cal62 and 15,000 TPC1 cells were used.

## Results

### Consequences of *ADAR1* knockdown on global gene expression

We previously demonstrated a key role for *ADAR1* in thyroid tumorigenesis [10]. Loss-of-function of *ADAR1* profoundly repressed proliferation, invasion, and migration in human thyroid tumor cell models and inhibited tumor growth in an in vivo xenograft model. Encouraged by these results, here we further investigated the function of *ADAR1* in thyroid cancer by examining the consequences of *ADAR1* knockdown on global gene expression. We performed RNA-seq in Cal62 thyroid cancer cells and compared the mRNA expression of siControl cells (transfected with a control siRNA) with those silenced for *ADAR1* using two independent siRNAs (ADAR1 #1 and ADAR1 #2). Of note, the two siRNAs employed targeted both the p150 and p110 *ADAR1* gene forms (Supplementary Figure 1).

**Fig. 1:**
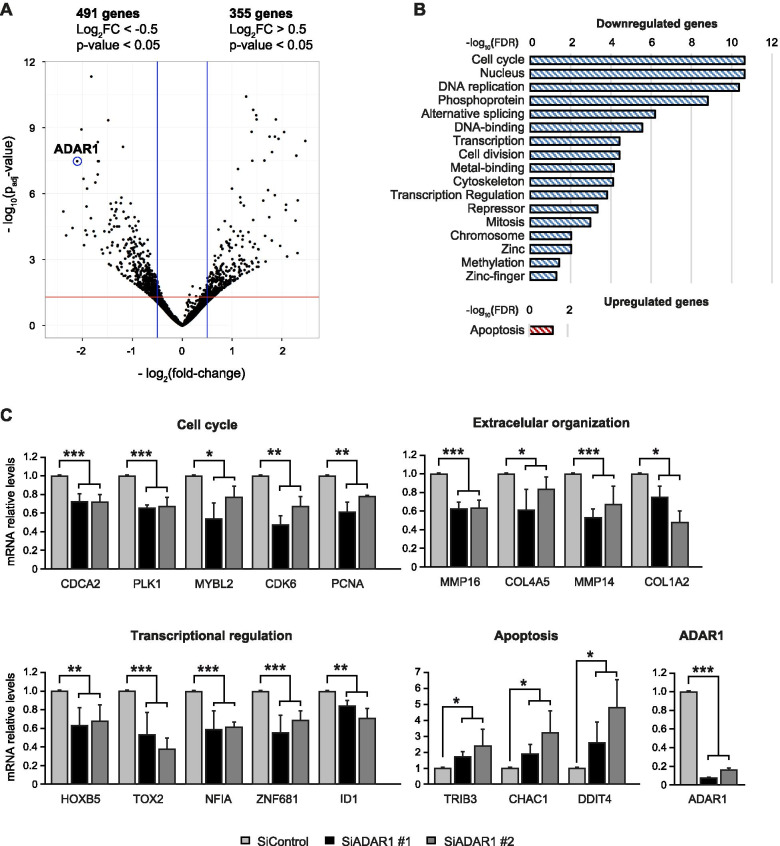
Effect of *ADAR1* knockdown on differential gene expression. **A** Volcano plot representing the RNA-seq differential expression analysis of Cal62 cells comparing siControl *versus* siADAR1 #1 and #2. *ADAR1* is highlighted in the figure among the downregulated genes. **B** Functional annotation for the down- and up-regulated genes. **C** RT-qPCR validation of dysregulated genes 72 h after *ADAR1* knockdown in Cal62 cells (*n* = 4). Error bars indicate standard deviations. Asterisks denote statistical significance compared with siControl treatment as assessed with Student’s t-test (two-tailed). * *p* < 0.05, ** *p* < 0.01, *** *p* < 0.001

We considered significant differences as those with an FDR-adjusted p-value < 0.05 and a log_2_ Fold Change (FC) < -0.5 for the downregulated events and a log_2_FC > 0.5 for the upregulated events. We found that 491 genes were downregulated and 355 genes were upregulated upon *ADAR1* knockdown (Fig. 1A). Reassuringly, *ADAR1* emerged as one of the main downregulated genes, confirming its knockdown in these cells (Fig. 1A). Functional annotation of the deregulated genes showed an enrichment of genes involved in cell cycle, DNA replication, cell division and cytoskeleton among the downregulated genes, and genes involved in apoptosis among the upregulated genes (Fig. 1B). These results fit well with the reduced proliferation, invasion and cell viability after *ADAR1* silencing in thyroid cancer models [10]. Some studies have previously implicated ADAR1 in alternative splicing regulation [3, 4] and, notably, genes involved in the alternative splicing process were well represented in our downregulated data set (Fig. 1B). Finally, genes associated with transcription and regulation were also enriched in the downregulated data set (Fig. 1B). We validated some of the RNA-seq results by qPCR in *ADAR1*-knockdown Cal62 cells; specifically, the highly significant candidate genes related to the aforementioned processes of cell cycle, extracellular organization, transcription regulation and apoptosis (Fig. 1C).

### ADAR1 has a major influence on global splicing patterns

**Fig. 2:**
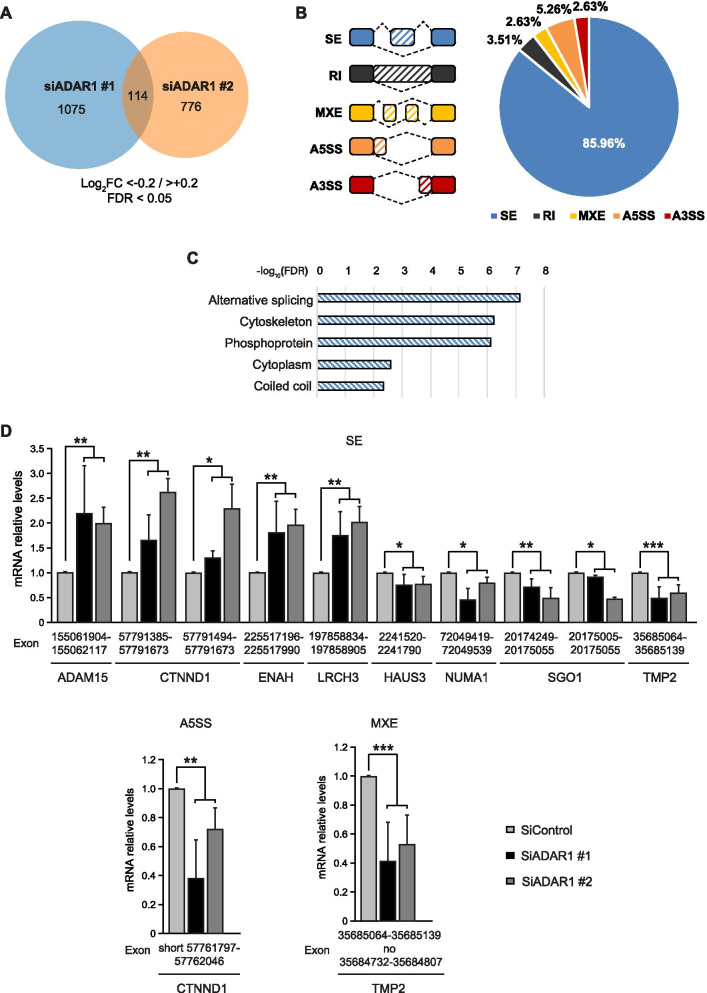
Consequences of *ADAR1* knockdown on alternative splicing. **A** Venn diagram representing the significant (FDR < 0.05 and Inclusion Level Difference < -0.2 / < 0.2) alternative splicing events differing between siControl and siADAR1 #1 (blue) or siADAR1 #2 (orange) in Cal62 cells. One hundred and fourteen events were common in both analyses. **B** Left panel: Schematic of the alternative splicing events analyzed. Right panel: Pie chart of events with significantly different inclusion levels (FDR < 0.05 and Inclusion Level Difference < -0.2 / < 0.2) upon *ADAR1* knockdown. Abbreviations: SE, skipped exon; RI, retained intron; MXE, mutually exclusive; A5SS, alternative 5’ splice site; A3SS, alternative 3’ splice site. **C** Functional annotation for the genes that present with significant differential alternative splicing events after *ADAR1* silencing. **D** RT-qPCR validation of ADAR1-regulated alternative splicing events for indicated genes 72 h after siRNA transfection in Cal62 cells (*n* = 4). Error bars indicate standard deviations. Asterisks denote statistical significance compared with siControl treatment as assessed with Student´s t-test (two-tailed). * *p* < 0.05, ** *p* < 0.01, *** *p* < 0.001

Given our results and the link between A-to-I RNA editing of pre-mRNAs by ADARs and splicing regulation [3, 4], we aimed to examine how ADAR1 editing globally influences alternative splicing. Several types of alternative splicing have been experimentally described [31], including skipped exons (SE), retained introns (RI) in the mature mRNA, mutually exclusive exons (MXE) and alternative 5’ or 3’ splice sites (A5SS or A3SS). We used the rMATS tool to determine differential alternative splicing [32] and filtered out events with < 15 total junction reads. We considered as significant events those with an FDR-adjusted p-value < 0.05 and an Inclusion Level Difference < -0.2 for downregulated events and > 0.2 for upregulated events. This analysis revealed 1075 high-confidence differential splicing events when comparing the siControl cells and the *ADAR1*-silenced cells using siRNA #1, and 776 high-confidence differential splicing events with the siRNA #2, with 114 differential alternative splicing events common in both comparisons (Fig. 2A). These results suggest that *ADAR1* silencing has an influence on the global splicing pattern of thyroid cancer cells. Among the alternative splicing types observed in the 114 common events, the majority were SE (85.96%) followed by A5SS (5.26%), RI (3.51%), MXE (2.63%) and A3SS (2.63%) (Fig. 2B). Regarding the affected splicing events, their inclusion was either repressed or promoted by ADAR1, although a general higher inclusion was observed in siADAR1 cells (data not shown). Functional annotation of the common alternately spliced genes revealed that the enriched genes were mainly involved in alternative splicing itself and in cytoskeleton processes (Fig. 2C). This latter finding may explain, at least in part, the suppression of migration and invasion observed in *ADAR1*-silenced cells [10]. As before, we experimentally validated the alternative splicing of selected candidate genes involved in cytoskeleton regulation by qPCR assays designed to distinguish between the spliced transcripts (Fig. 2D).

### ADAR1 edits hundreds of transcripts in thyroid cancer cells

**Fig. 3:**
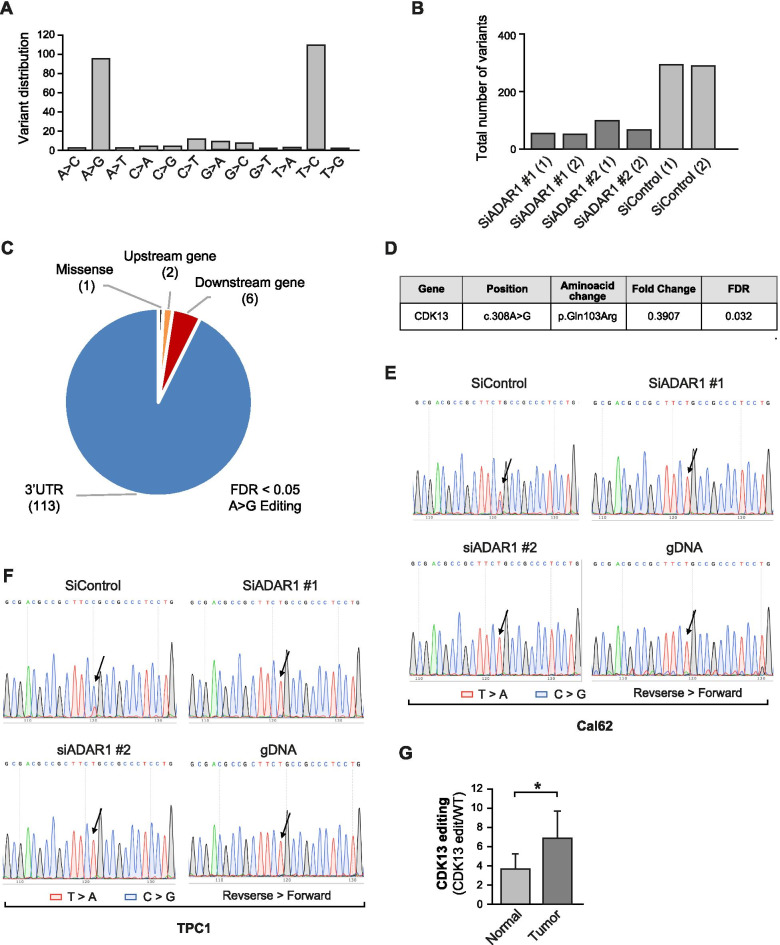
ADAR1 edits *CDK13* transcript at position c.308A > G. **A** Distribution of 12 types of nucleotide changes across the entire transcriptome of Cal62 cells after *ADAR1* knockdown, as profiled by RNA-seq. **B** RNA-seq analysis showing a global reduction in the total number of variants after *ADAR1* silencing. **C** Distribution of A-to-I editing sites over annotated genomic regions. Abbreviations: UTR, untranslated region. **D** Details of the position and significance of the *CDK13* editing event that is downregulated after *ADAR1* knockdown in Cal62 cells. Fold-change and FDR refers to the allele frequency change in siControl *versus* siADAR1. **E** and **F** Sanger sequencing chromatograms using a reverse primer illustrate editing of the selected *CDK13* event in siControl, siADAR1 #1, siADAR1 #2 and genomic DNA (gDNA) in Cal62 (**E**) and TPC1 (**F**) thyroid cancer cell lines. **G** CDK13 c.308 A > G editing frequency in normal (*n* = 6) and PTC tumor samples (*n* = 6) calculated by taking the peak area of G *versus* A using Indigo. Error bars indicate standard deviations. Asterisks denote statistical significance as assessed with Student’s t-test (two-tailed). * *p* < 0.05

We next analyzed the editing profile under the same conditions. A-to-I editing occurs at the level of RNA, but when the RNA is reverse transcribed to complementary DNA the inosine is converted to guanine. This A-to-G conversion, which is evidence of RNA editing, can be identified by comparing cDNA sequences with the reference genome. Global single nucleotide variant analysis revealed an enrichment in A>G or complementary T>C changes in *ADAR1*-silenced cells, which is to be expected since ADAR1 is responsible for A-to-I editing (Fig. 3A). Moreover, the total number of variants was dramatically repressed by silencing of *ADAR1* (Fig. 3B). These data confirm that *ADAR1* knockdown reduces global editing activity. After SNP filtering, 122 A-to-I editing events (FDR < 0.05) were found in the transcriptome of the siControl cells and were markedly lower in siADAR1 #1 and #2 cells (FC > 0). Based on location, the RNA editing sites could be divided into different categories: 3’ untranslated region (UTR), 5’UTR, upstream and downstream gene regions, missense, nonsense or synonymous. We identified 113 RNA editing sites in 3’UTRs, 6 in downstream and 2 in upstream gene regions, and 1 missense variant (Fig. 3C). Notably, most of the genes that showed a dissimilar alternative splicing pattern were not found among the edited transcripts. This phenomenon has been previously observed [3, 4] and suggests that the regulation of splicing directly by alteration of splicing-related motifs may not be the sole mechanism by which ADAR1 mediates splicing regulation. RNA editing events within transcripts of genes related to the splicing machinery or editing-independent ADAR1 regulation may also globally affect the splicing of many transcripts.

### *CDK13* is edited in thyroid cancer cells

We focused on the editing event that produced the one missense mutation because of its potential impact on the resulting protein by amino acid substitution, affecting protein conformation, localization and/or interactions. This editing event occurred in the *CDK13* transcript, a gene related to splicing [19, 20] and transcription [18] (Fig. 1B). Of note, the involvement of *CDK13* in the splicing process could explain, at least in part, the effect of ADAR1 on the observed global splicing patterns (Fig. 2). The editing site was located at position 308 (c.308A > G) on the cDNA, and resulted in an amino acid change from glutamine to arginine at position 103 (Q103R) of the CDK13 N-terminal region (Fig. 3D). To confirm *CDK13* editing in the Cal62 cell line used for RNA-seq, we performed Sanger sequencing for both cDNA (obtained directly from RNA) and genomic DNA (gDNA). As shown in Fig. 3E, a high frequency of *CDK13* RNA editing was detected in siControl cells but not in equivalent *ADAR1*-silenced cells or in the gDNA sequence. Identical results were obtained in a second thyroid cancer cell line, TPC1, in which the edited form was even more abundant than the WT form in control cells (Fig. 3F). Thus, the CDK13 c.308A > G editing event is present in thyroid tumor cells and is dependent on ADAR1 expression.

Importantly, when we analyzed the WT and edited forms of *CDK13* by RT-PCR and Sanger sequencing in PTC and contralateral normal tissue from 6 patients, we found a significantly higher level of edited *CDK13* in the tumor tissue samples (Fig. 3G). These data suggest that *CDK13* editing also occurs in thyroid tissue and that, in accordance with the high A-to-I editing activity observed in thyroid cancer [7, 8], a higher editing rate of *CDK13* is evident in tumor samples. While we acknowledge that the number of thyroid tumor samples analyzed is small, these data support the in vitro observations and indicate that the editing event is a significant phenomenon.

### *CDK13* silencing reduces proliferation, viability and invasion in thyroid cancer cells

**Fig. 4:**
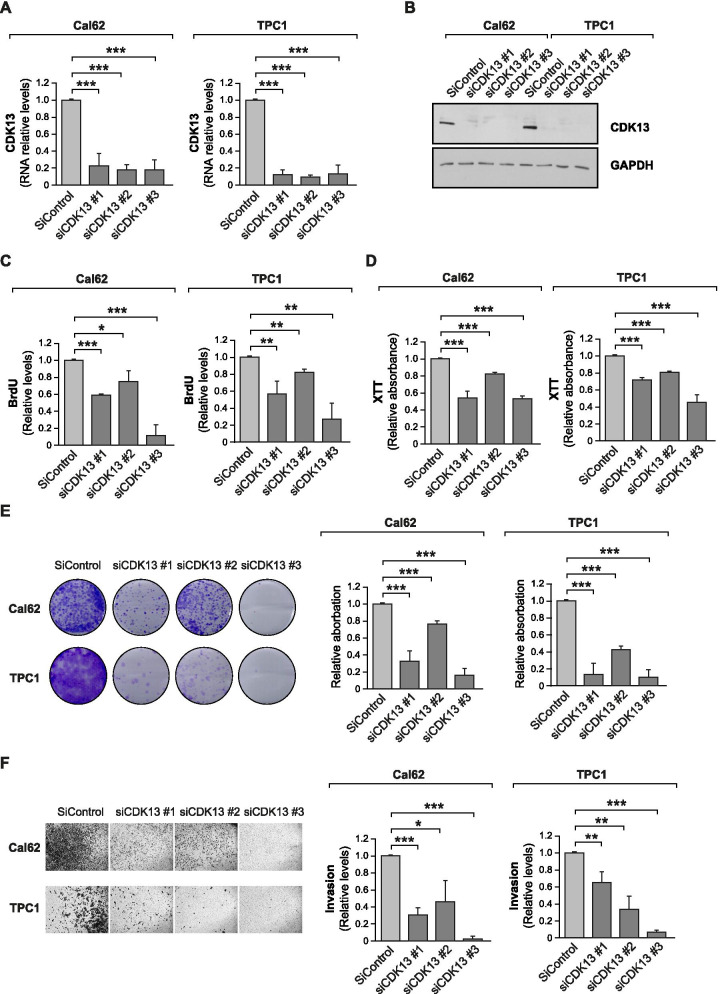
CDK13 is functionally important for thyroid cancer development. Cal62 and TPC1 cells were transfected with three independent siRNAs against *CDK13* or a control siRNA (siControl). **A** *CDK13* RNA relative levels (*n* = 3). **B** Representative western blotting for CDK13. GAPDH was used as loading control. **C** Proliferation by BrdU incorporation (*n* = 3). **D** and **E** Viability assays tested by XTT dye reduction (*n* = 3) (**D**) or crystal violet staining (*n* = 4) (**E**). **F** Matrigel Transwell invasion assay (*n* = 3). Error bars indicate standard deviations. Asterisks denote statistical significance compared with siControl treatment as assessed with Student’s t-test (two-tailed). * *p* < 0.05, ** *p* < 0.01, *** *p* < 0.001

There are few studies on the role of *CDK13* in cancer [33, 34] and, to our knowledge, none of them have addressed its role in thyroid cancer. To examine the function of *CDK13* and how its expression affects the main cancer hallmarks in thyroid cancer, we performed loss-of-function assays using three independent siRNAs that markedly decreased *CDK13* RNA and protein levels in Cal62 and TPC1 thyroid cancer cells (Fig. 4A, B). We observed that *CDK13* silencing inhibited cell proliferation, as measured by BrdU incorporation (Fig. 4C), and cell viability, as measured by XTT dye reduction and crystal violet staining (Fig. 4D–E). Notably, *CDK13* knockdown suppressed cell invasion (Fig. 4F), a fundamental hallmark for cancer progression and dissemination, pointing to an oncogenic role for *CDK13* in thyroid cancer cells.

### Edited CDK13-Q103R promotes aggressive phenotypes of thyroid cancer cells

**Fig. 5:**
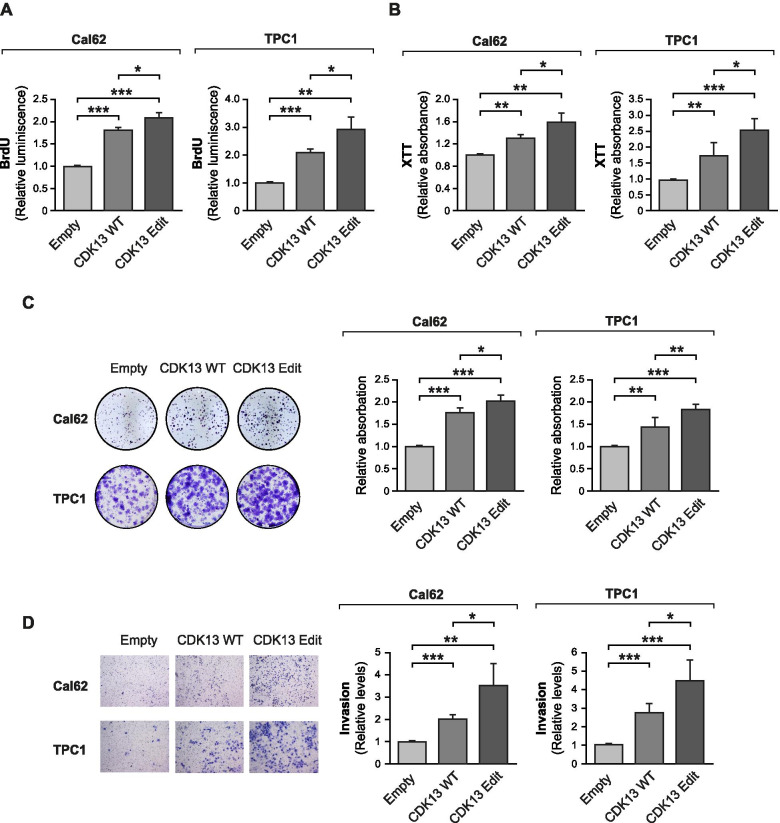
Edited *CDK13* potentiates the aggressive behavior of thyroid cancer cells. Cal62 and TPC1 cells were stably infected with lentiviruses containing the WT CDK13 or its edited form (Edit CDK13) and were assayed for (**A**) BrdU incorporation (*n* = 3). **B** XTT dye reduction (*n* = 3 for Cal62 and *n* = 5 for TPC1) and (**C**) crystal violet staining (*n* = 4). **D** Matrigel Transwell invasion assay (*n* = 4). Error bars indicate standard deviations. Asterisks denote statistical significance compared with siControl treatment as assessed with Student’s t-test (two-tailed). * *p* < 0.05, ** *p* < 0.01, *** *p* < 0.001

We further investigated CDK13 because of its oncogenic behavior in thyroid cancer cell lines (Fig. 4C–F) and because an edited form of its transcript is dominant in primary thyroid tumors (Fig. 3G). We hypothesized that an amino acid change in the protein could be relevant for its tumorigenic behavior in thyroid cancer cells. To test this, we established lentiviral constructs to overexpress WT-CDK13 or its edited c.308A > G (Q103R) form (edit-CDK13). We transduced both Cal62 and TPC1 cells and obtained stable cell lines with constitutive overexpression of the CDK13 forms tagged with HA, or an empty vector control cell line (Supplementary Figure 2A, B). In accord with the loss-of-function assays, we observed that WT-CDK13 stimulated proliferation (Fig. 5A), cell viability (Fig. 5B, C) and invasion (Fig. 5D) in Cal62 and TPC1 cells. Remarkably, the aforementioned features were significantly greater in edit-CDK13 cells than in WT-CDK13 cells (Fig. 5A–D). These results demonstrate that the ADAR1 editing event in the *CDK13* transcript potentiates the aggressive behavior of the thyroid cancer cells.

### Edited *CDK13* rescues the proliferation and invasion defects in ADAR1-silenced cells

**Fig. 6:**
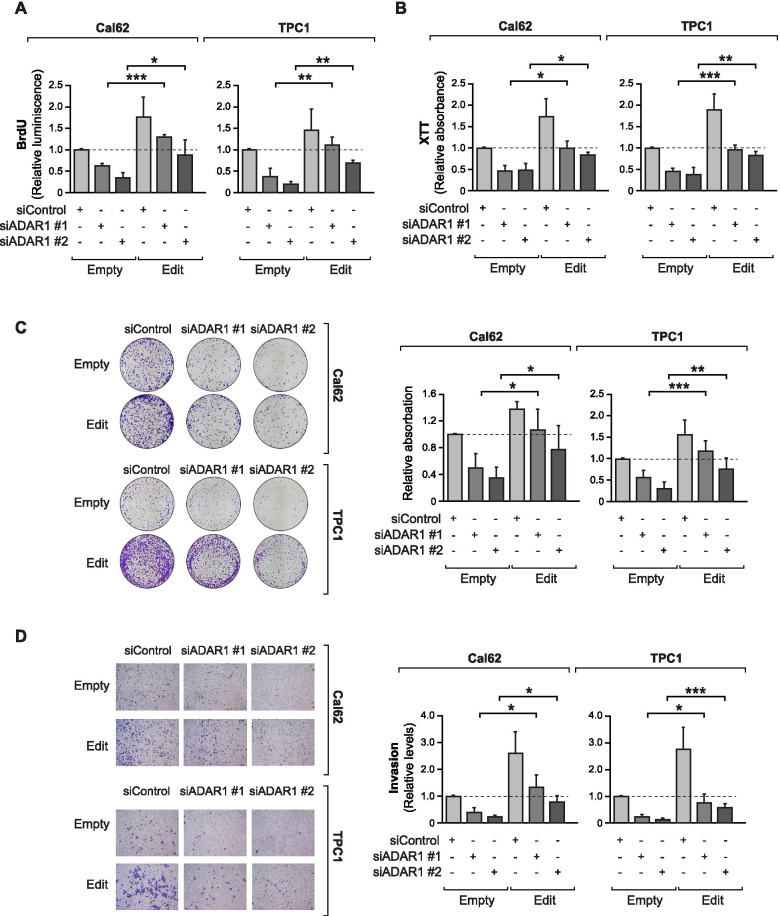
Edited *CDK13* rescues the *ADAR1*-knockdown phenotype. Stably-transduced Cal62 and TPC1 cells with CDK13 Edit or Empty vector were silenced for *ADAR1* and were assayed for (**A**) BrdU incorporation (*n* = 4 for Cal62 and *n* = 3 for TPC1 cell line). **B** and **C** Viability by XTT dye reduction (*n* = 3) (**B**) and crystal violet staining (*n* = 5) (**C**). **D** Matrigel Transwell invasion assay (*n* = 3 for Cal62 and *n* = 4 for TPC1 cell line). Error bars indicate standard deviations. Asterisks denote statistical significance compared with siControl treatment as assessed with Student’s t-test (two-tailed). * *p* < 0.05, ** *p* < 0.01, *** *p* < 0.001

To assess the importance of the *CDK13* editing event in the oncogenic function of ADAR1 we overexpressed the edited form of CDK13 in thyroid cancer cells that were simultaneously silenced for *ADAR1* using two different siRNAs (Supplementary Figure 3A, B). We observed that the suppressive effect of *ADAR1* knockdown on proliferation (Fig. 6A), viability (Fig. 6B, C) and invasion (Fig. 6D) was rescued by the overexpression of edit-*CDK13* in Cal62 and TPC1 cells. Of note, when WT-CDK13 was overexpressed as a control, we observed that in contrast to the complete rescue achieved by the edited form, the decrease in cell viability induced by *ADAR1* silencing was not reversed (Supplementary Figure 3C). Moreover, the two cancer cell lines exhibited a different CDK13 WT/edit ratio, with a higher representation of the edited form in TPC1 cells (Fig. 3F) and higher levels of CDK13 WT in Cal62 cells (Fig. 3E). This indicates that the outcomes are independent of the endogenous CDK13 WT/edited levels, and suggests that CDK13 editing has a relevant role in the oncogenic function of *ADAR1* in thyroid cancer.

### *CDK13* editing changes the nuclear localization of the protein

CDK13 is a member of the cyclin-dependent serine/threonine protein kinase family, with a conserved central kinase domain in the C-terminal region and a regulatory N-terminal region. The N-terminal region contains arginine-serine (RS)-rich domains (Supplementary Figure 4A) that differentiate this kinase from other family members. The RS-rich domains span residues 200–435 in CDK13 [18] and link this kinase to the serine-arginine (SR) protein family, which are required for both constitutive and alternative pre-mRNA splicing. In addition, the N-terminal region contains three bipartite nuclear localization signals (NLS) that determine the nuclear localization of the protein [20]. As the c.308A > G edited site is located in position 103 of the CDK13 N-terminal region (Supplementary Figure 4A), we speculated that this amino acid change might affect the NLS sequences and, consequently, CDK13 localization. Using NLS mapper [35], we found that the amino acid change alters four bipartite NLS sequences in the N-terminal region and increases the predicted scores, indicating stronger NLS activity (Supplementary Figure 4B). We assessed CDK13 localization by protein fractionation and western blotting and observed that both WT and edited forms of CDK13 were localized to the nucleus (Supplementary Figure 4C).

**Fig. 7:**
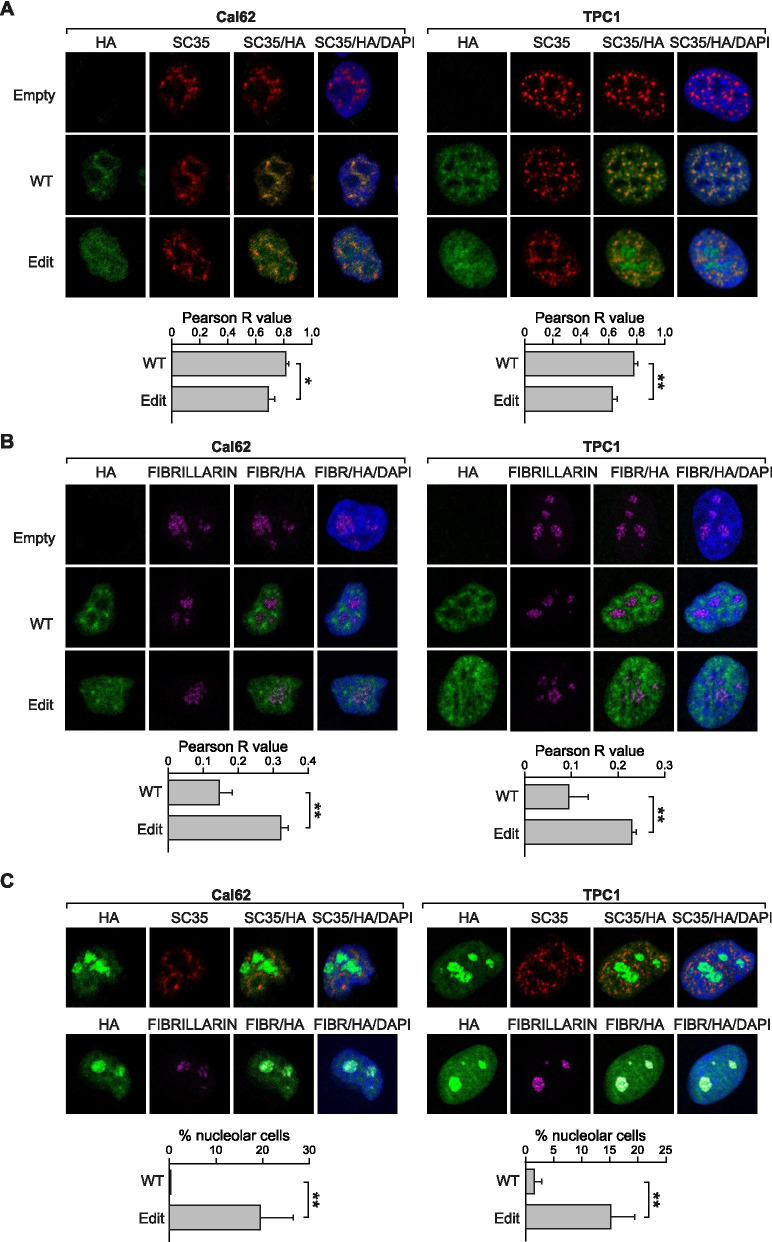
Edited and wild-type CDK13 show differences in nuclear speckle and nucleolus localization. Stably-transduced CDK13-WT, CDK13-Edit or Empty vector Cal62 and TPC1 cell lines were used. **A** and **B** Confocal images of immunofluorescence staining of the cells with antibodies against HA (green), the nuclear speckle marker SC35 (red) (**A**) or the nucleolus marker Fibrillarin (Pink) (**B**). Nuclei were stained with DAPI (blue). Colocalization was analyzed using Fiji-Coloc2 plugin, and the mean of the Pearson R value from 3 independent experiments is represented in the bottom panels. **C** Upper panels: Representative image of CDK13-Edit Cal62 and TPC1 cells with nucleolar accumulation of CDK13. The images represent the immunofluorescence staining of cells with antibodies against HA (green), SC35 (red) or Fibrillarin (pink). Bottom panel: Graphs show quantification of the percentage of CDK13-WT or CDK13-Edit cells that present with nucleolar accumulation of CDK13 (*n* = 3 independent experiments). Error bars indicate standard deviations. Asterisks denote statistical significance as assessed with Student’s t-test (two-tailed). * *p* < 0.05, ** *p* < 0.01, *** *p* < 0.001

Even et al*.* [20] have demonstrated that CDK13 can shuttle between the nucleoplasm and nuclear speckles, and that the N-terminal region is critical for localization of the kinase in these structures. Nuclear speckles are nuclear domains enriched in pre-mRNA splicing factors, located in the interchromatin regions of the nucleoplasm of mammalian cells [36]. We used immunofluorescence to analyze the localization of the WT and edited forms of CDK13 in transduced Cal62 and TPC1 cells. We also used an antibody directed against the splicing factor SC35, which specifically marks nuclear speckles [20]. We observed that WT-CDK13 correlated highly with the nuclear speckles in thyroid cancer cells (Fig. 7A), suggesting that the described role of CDK13 in splicing [19, 20] is maintained in thyroid cells. By contrast, edit-CDK13 showed a significantly lower correlation with nuclear speckles in Cal62 and TPC1 cells (Fig. 7A). In addition, we observed that edit-CDK13 was more uniformly expressed in the nucleus, whereas WT-CDK13 was almost nonexistent in some patches, likely corresponding to the nucleolus. To validate this, we utilized an antibody against the nucleolar protein Fibrillarin, and observed that WT-CDK13 was absent from the nucleolus, whereas edit-CDK13 showed a strong co-localization with this structure (Fig. 7B). Notably, 19.6% of Cal62 and 15.2% of TPC1 cells transduced with edit-CDK13 showed enrichment of CDK13 at the nucleolus, as demonstrated by co-localization with Fibrillarin, but not at the nuclear speckles (Fig. 7C). When the same experiment was performed with WT-CDK13, no accumulation was found in the nucleolus in Cal62 cells and only 1.5% of TPC1 cells showed nucleolar accumulation. These findings demonstrate that editing of *CDK13* induces a change in the localization pattern of the protein, decreasing its association with nuclear speckles but enriching its localization at the nucleolus.

### *CDK13* editing induces changes in splicing patterns

**Fig. 8:**
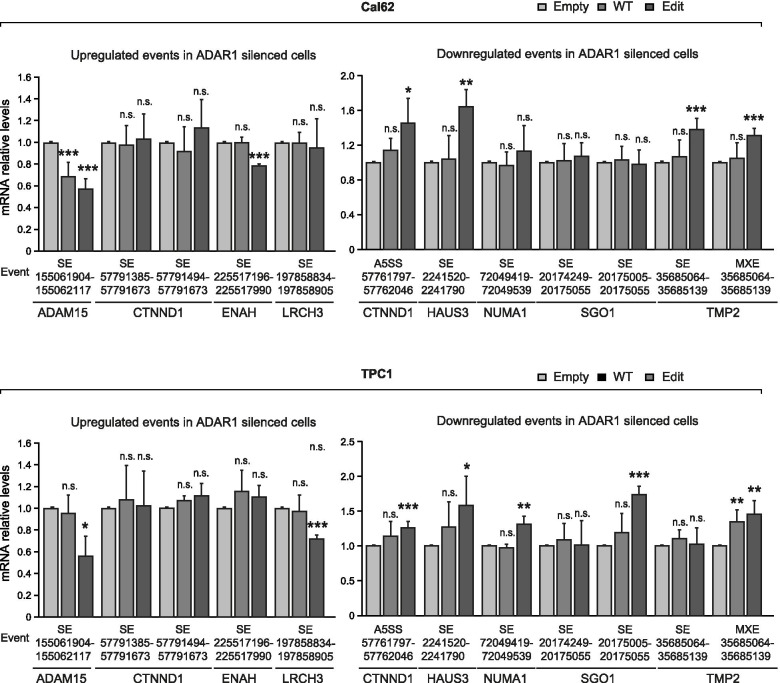
Edited *CDK13* alters alternative splicing. RT-qPCR of ADAR1-regulated alternative splicing events for indicated genes in Cal62 (*upper panel*) or TPC1 cells (*lower panel*) stably expressing CDK13-WT, CDK13-Edit or the empty vector (*n* = 4). Error bars indicate standard deviations. Asterisks denote statistical significance compared with siControl treatment assessed by Student’s t-test (two-tailed). * *p* < 0.05, ** *p* < 0.01, *** *p* < 0.001

Finally, given the described involvement of CDK13 in the splicing process [19, 20], we questioned whether the change in *CDK13* editing would contribute to some of the *ADAR1*-silencing-induced changes in splicing highlighted in our RNA-seq data. We analyzed the levels of the candidate splicing products altered in *ADAR1*-silenced cells; specifically, those potentially involved in the cytoskeleton and later validated by qPCR. We observed that some splicing products were increased (A5SS event involving the exon 57,761,797–57,762,046 in *CTNND1*, SE of exon 2,241,520–2,241,790 of *HAUS3*, and the MXE splicing event involving the exon 35,685,064–35,685,139 in *TMP2*) or decreased (SE 155,061,904–155,062,117 in *ADAM15*) following transfection of edit-CDK13 in both Cal62 and TPC1 cells (Fig. 8). Some splicing events were not consistently altered in both cell lines such as the SE events in *NUMA1, SGO1* and *LRCH2* in TPC1 cells, or the SE in *TMP2* and *ENAH* in Cal62 cells (Fig. 8). As expected, the splice products increased in edit-CDK13 cells were lower in *ADAR1*-silenced cells, where the edited form of *CDK13* is almost absent, and vice versa. Interestingly, we observed a different pattern of splicing events in cells overexpressing WT and edited CDK13, with the transduction of the edited form responsible for most of the alternative splicing changes.

## Discussion

Post-transcriptional modification of RNA is a wide-spread phenomenon that expands the transcriptome and the range of functions of RNA transcripts. One of the most surprising discoveries in this field is that mammalian RNAs can undergo RNA editing, which modifies specific RNA nucleotides without altering the DNA template [37, 38]. A-to-I editing can profoundly influence cellular functions by altering mRNA splicing, stability, localization, and translation, and by interfering with the binding of regulatory RNAs [39]. The magnitude and the biological consequences of A-to-I editing in the majority of cancers remain largely unknown.

Advances in high-throughput sequencing and data generation have revealed that RNA editing events are extensive across the human cancer transcriptome, and that the incidence and progression of multiple cancers are associated with some of these events [7, 8, 39–41]. In the present study, we aimed to assess the impact of RNA editing in thyroid cancer and to identify de novo cancer-related RNA editing sites using next generation sequencing in an *ADAR1*-knockdown cellular model. We believe that thyroid cancer represents a suitable model for this study as RNA editing activity is enriched in thyroid tumors over normal tissue [7, 8] and because we previously demonstrated the profound functional consequences of *ADAR1* knockdown for tumor progression [10]. To the best of our knowledge, no other study has systematically studied RNA editing in thyroid cancer. Our transcriptome analysis revealed novel changes triggered by ADAR1 deregulation in almost one thousand transcripts, most of them involved in fundamental processes for cancer development and progression such as cell cycle, DNA replication, regulation of the cytoskeleton and apoptosis. This finding is consistent with our previous results that functionally associated ADAR1 expression with proliferation, invasion, migration and in vivo tumor growth [10]. Moreover, *ADAR1* silencing induced the downregulation of several oncogenes and the upregulation of tumor suppressor genes [10], confirming the oncogenic role of *ADAR1* in thyroid cancer observed in other cancer types [42–47]. Beyond its oncogenic activity, ADAR1 has been shown to elicit an important role in immunity [48, 49], and mutations or alterations in ADAR1 can confer autoimmunity in humans and in animal models [49–54]. Along this line, our RNA-seq results reveal that the innate immune response (GO: 0,045,087) and cytokine production (GO: 0,001,817) are biological processes enriched upon *ADAR1* silencing (data not show). Because autoimmune thyroid disease is closely related to thyroid cancer [55], it is becoming increasingly important to identify potential diagnostic biomarkers and therapeutic targets for both diseases and ADAR1 could be a good candidate. Future studies should be orientated in this direction.

The deregulation of genes involved in immunity is likely elicited by the silencing of the p150 ADAR1 isoform. However, other biological processes related to the p110 isoform [56], also silenced in our study, were deregulated, such as DNA damage checkpoint (GO:0,000,077). It is highly probable that both ADAR1 isoforms contribute to the growth defect observed in vitro and in vivo after *ADAR1* silencing [57]; however, our study design did not allow us to distinguish the specific isoform responsible for CDK13 editing and this should be addressed in a future study.

Interestingly, alternative splicing was enriched among the deregulated genes after *ADAR1* silencing. Detailed analysis revealed that *ADAR1* has a prominent influence on the global splicing pattern and identified approximately one hundred high-confidence splicing events affected by ADAR1. These gene sets are enriched with similar functions, for example, we observed several genes involved in the cytoskeleton and thus, likely contributing to the altered invasion, migration or 3D growth previously observed upon ADAR1 loss-of-function [10]. Other studies have also provided evidence for crosstalk between RNA editing enzymes and the splicing machinery [3, 4, 58]. Nevertheless, detailed mechanistic explanations and their biological importance in cancer are lacking. Regulation of alternative splicing involves *cis*-acting elements within the pre-mRNAs, and *trans*-acting factors such as SR proteins and heterogeneous nuclear ribonucleoproteins [3, 59–63]. RNA-seq analysis demonstrated that most of the transcripts with significantly altered splicing did not present with changes in edited adenosines, suggesting that the ADAR1-dependent regulation of alternative splicing is not only dependent on the direct editing of *cis*-acting elements. Other studies have obtained comparable results in other cell types, with few alternately spliced sites explained by nearby A-to-I edit sites [3, 4]. Our identification of an editing event in *CDK13*, which encodes for an SR-related protein implicated in splicing [19, 20], might shed some light on this phenomenon. We propose that the extent of ADAR1-regulated splicing is mediated predominantly by trans-acting elements like CDK13, rather than direct *cis*-element editing, leading to a broad influence on splicing for many genes. Indeed, our results demonstrate that *CDK13* editing is responsible for some of the splicing events identified by our RNA-seq analysis. Interestingly, genes showing significant changes in their splicing pattern are frequently involved in the splicing activity itself. This has been also been observed in hepatocellular carcinoma, myelogenous leukemia and glioblastoma [3]. This may suggest that a first editing event that influences the splicing process, such as *CDK13* editing, could produce splicing changes of other alternative splicing regulators, ultimately having a prominent influence on the global splicing pattern observed.

We believe that our results obtained in thyroid cancer cells and tumor samples can be extrapolated to other cancer types. *ADAR1* and *CDK13* have been demonstrated to act as oncogenes in several cancer types [33, 34, 42, 64–66]. Moreover, in agreement with the oncogenic behavior conferred by *CDK13* editing described herein, the *CDK13* editing event (c.308A > G) has been found to be overrepresented in several tumor types as compared with normal control samples, such as hepatocellular carcinoma [67], glioblastoma [68] and kidney renal clear cell carcinoma [7]. Supporting our results in thyroid cancer, *CDK13* editing correlated with poor prognosis in hepatocellular carcinoma [67]. Moreover, we observed that this editing event is present in the triple-negative breast cancer cell lines BT20 and MB468, and is also dependent on ADAR1 expression (our unpublished observations). We believe that this is particularly relevant as ADAR1 and CDK13 have both been described as important factors and possible therapeutic targets in triple-negative breast cancer [33, 69].

To our knowledge, this is the first study to analyze the functional importance of *CDK13* editing in cancer. Few studies had previously demonstrated the function of CDK13 in tumorigenesis. CDK13 was described as amplified in hepatocellular carcinoma and exhibits oncogenic activity [34]. Other studies showed that the simultaneous inhibition of CDK12 and CDK13 suppresses tumorigenic features in leukemia [64], ovarian cancer [65] and triple-negative breast cancer [33]. However, Quereda et al. [33] were the only group to demonstrate that downregulation of CDK13 (without simultaneous CDK12 inhibition) inhibited colony formation in a breast cancer cell line. Our functional analysis shows that, in accordance with previous studies [33, 34, 64, 65], *CDK13* acts an oncogene in thyroid cancer cells, identifying it as a new therapeutic target in thyroid and other cancer types. The fact that TCGA data show no *CDK13* upregulation in PTC would indicate that the mechanism of CDK13 oncogenic activation in PTC is via editing rather than upregulation. As we observed, edited Q103R CDK13 stimulates a stronger cell proliferation and migration phenotype than WT CDK13 and, probably because of this, *CDK13* editing might be an efficient mechanism contributing to tumor progression and aggressiveness.

In summary, our work confirms *ADAR1* as an oncogene in thyroid cancer and offers evidence of its mechanism of action through editing *CDK13*, which confers stronger tumorigenic properties to cells and delocalizes CDK13 from nuclear speckles, the main hub for splicing factors. Moreover, editing of this SR-related protein changes the splicing of several transcripts and may explain, at least in part, the splicing pattern induced by ADAR1 deregulation. This work opens the door for detailed studies of the mechanism of action of CDK13 in thyroid cancer with respect to how edited CDK13 triggers the observed oncogenic behavior. In this line, we observed a change in the splicing pattern of some potentially interesting candidate genes. For example, *ADAM15* splicing is altered after edit-CDK13 overexpression in both Cal62 and TPC1 cell lines. This is of special relevance because aberrant *ADAM15* expression has been associated with human cancer [70] and the alternative splicing of *ADAM15* is mis-regulated in cancer cells [71]. Indeed, the splicing form upregulated in edit-CDK13 cells has been associated with poor prognosis in patients with node-negative breast cancer [72]. Other interesting candidates previously related to cancer include *CTNND1* [73] and *TPM2* [74, 75], for which alternative splicing events in both transcripts have been correlated with cancer [76, 77], and *HAUS3* [78].

Finally, our work supports ADAR1-mediated A-to-I editing as an important pathway in cancer progression, and highlights *ADAR1*, *CDK13* and the edited CDK13-Q103R as potential targets for the development of new treatments for thyroid and other cancer types.

## Supplementary Information

**Additional file 1: Table S1.** Primers

**Additional file 2: Supplementary Figure 1.***ADAR1* silencing. Representative western blot of ADAR1 steady-state expression at the indicated time points after *ADAR1* silencing in Cal62 and TPC1 cell lines. GAPDH was used as a loading control.

**Additional file 3: Supplementary Figure 2.** CDK13 levels in cells stably expressing CDK13-WT, CDK13-Edit or the Empty vector. Cal62 and TPC1 were infected with lentivirus expressing the WT or the edited form of *CDK13* (CDK13-WT and CDK13-Edit, respectively) or the corresponding empty vector, and selected using puromycin. (A) *CDK13* levels in the indicated cell lines. (B) Representative western blotting for HA antibody in the indicated cell lines. Vinculin was used as loading control (*n* = 3). Error bars indicate standard deviations. Asterisks denote statistical significance compared with siControl treatment assessed by Student’s t-test (two-tailed). n.s: non significative p > 0.05, ** *p *< 0.01, *** *p* < 0.001.

**Additional file 4: Supplementary Figure 3.** CDK13 overexpression and *ADAR1* silencing in Cal62 and TPC1 cell lines. RNA relative levels for *CDK13* (A) and *ADAR1* (B) in Cal62 and TPC1 cell lines stably expressing CDK13-Edit 72 hours after *ADAR1* siRNA (siADAR1 #1 and #2) or Control siRNA (siControl) transfection. (C) Stably-transduced Cal62 and TPC1 cells with CDK13-Edit, CDK13-WT or empty vector were silenced for *ADAR1* and were assayed for cell viability by XTT dye reduction (*n* = 4). Asterisks denote statistical significance assessed by Student’s t-test (two-tailed). n.s: non significative *p* > 0.05, ** *p *< 0.01, *** *p* < 0.001.

**Additional file 5: Supplementary Figure 4.** Both CDK13-WT and CDK13-Edit are located in the cell nucleus. (A) Schematic representation of the CDK13 domain structure and the CDK13 editing event. Numbers below indicate the amino acid position. Abbreviations: RS: arginine/serine-rich; KD: kinase domain; C: C-terminal extension. (B) Predicted bipartite NLS (Nuclear Localization Sequence) in the WT and c.308 A>G edited form of CDK13. Predictions were performed using NLS mapper (http://nls-mapper.iab.keio.ac.jp). (C) Representative western blotting for HA and CDK13 in the indicated Cal62 and TPC1 cells after cytoplasm-nucleus fractionation. SP1 and tubulin were used as loading control for the nucleus and the cytoplasm, respectively.

## Abbreviations

A3SS: 3’ Splice sites
A5SS: 5’ Splice sites
ADAR1: Adenosine deaminases acting on RNA
ATC: Anaplastic Thyroid Carcinoma
BrdU: Bromodeoxyuridine
CDK13: Cyclin-Dependent Kinase 13
FDR: False discovery rate
FTC: Follicular Thyroid Carcinoma
GAPDH: Glyceraldehyde Phosphate Dehydrogenase
GEO: Gene Expression Omnibus
MATS: Multivariate Analysis of Transcript Splicing
MXE: Mutually exclusive exons
NLS: Nuclear Localization Signals
PBS: Phosphate-Buffered Saline
PDTC: Poorly Differentiated Thyroid Carcinoma
PTC: Papillary Thyroid Carcinoma
qRT-PCR: Quantitative real-time PCR
RI: Retained introns
RNA-seq: RNA-sequencing
SDS-PAGE: Sodium Dodecyl Sulfate–Polyacrylamide Gel Electrophoresis
SE: Skipped exons
SNP: Single Nucleotide Polymorphism
TCGA: The Cancer Genome Atlas
TPM: Transcript Per million
UTR: Untranslated region
WT: Wild-type
