# Supplementary material for: An ADAR1-dependent RNA editing event in the cyclin-dependent kinase CDK13 promotes thyroid cancer hallmarks
Source: Mol Cancer. 2021 Sep 8;20:115. doi: 10.1186/s12943-021-01401-y (PMC8424981; doi:10.1186/s12943-021-01401-y)
Supplement: Supplementary file 1 — Additional file 1: Table S1. Primers [file 12943_2021_1401_MOESM1_ESM.pdf]

Table S1. Primers

Coding genes:

Name	Forward	Reverse
CDCA2	CCGAAGACTGGGTTCAGGTTA	TTGCCAAGCGATGAGGAACT
PLK1	CCGCAATTACATGAGCGAGC	TGAGCTTGGTGTGATCCTGG
MYBL2	GGCCAGCCACTTCCCTAACC	TCAGGTGCTTGGCAATCAGT
CDK6	GGAGTGCCCACTGAAACCAT	CGTGACGACCACTGAGGTTA
PCNA	TCTGAGGGCTTCGACACCTA	TTCTCCTGGTTTGGTGCTTCA
MMP16	GCTCGTCCATCCATTGAAGC	TGTAACCAAACCTCCACATTGA
COL4A5	CACGGTCAAGACTTGGGGAC	CTCTGGCCCTTTAGGGGTTG
MMP14	CTTTATGGGGGTGAGTCAGGG	GGTTATTCCTCACCCGCCAG
COL1A2	TGTGGATACGCGGACTTTGT	CAGCAAAGTTCCCACCGAGA
HOXB5	ACCGAAATAGACGAGGCCAG	GCCCGGTCATATCATGGCT
TOX2	CCACGGCGGCAAGTTTGAT	GAGTGGTAGCTGGCTTCGTG
NFIA	AGTGGCAGTCAGTCAAGTGG	TTGCATGCGGACTTGCTCT
ZNF681	TGGCAATGCCTGGACACTAT	ACAAATAACTGGGGGTTCGG
ID1	AGGTGAGCAAGGTGGAGATTC	AACGCATGCCGCCTCG
TRIB3	GCGCGGCCAGATGCGA	AGTTGCACGATCTGGAGCAG
CHAC1	TCCTTGAAGATCATGAGGGCTG	TGCCTTCAGTGGTTGGTCAG
DDIT4	CTTTGGGACCGCTTCTCGT	ATCCAGGTAAGCCGTGTCTTC
ADAR1	CATCAGCGGGCTGTTAGAAT	CTTGGCCACTTTCTTGCTTC
GAPDH	TGCACCACCAACTGCTTAGC	GGCATGGACTGTGGTCATGAG
CDK13	GGGACTACTGGAATCAGGCTT	ATACGGCCGACTTTCTTCTGA

Splicing events:

Name	Forward	Reverse
ADAM15 SE 155061904-155062117	AGGCACTAAGGCTGAGCTG	AGAGGCCGTGCCCTTCTCT
CTNND1 SE 55791385-57791673	CACATTTGAGTGTGAAGTGAGG	AGTCGTCCATGAAGGTAAGG
CTNND1 SE 57791494-57791673	CACATTTGAGGTGGAGTCGA	TTGGCATCTTGTGGTGAGAC
ENAH SE 225517196-225517990	TCAAGTGCTGCTCCATCTTC	GAGCTGAGATGACTTTAGCG
HAUS3 SE 2241520-2241790	ACTTCCTGAGCGTGCTTAGG	TTCAGCCAGCGCTGAGGCA
LRCH3 SE 197858834-197858905	CTTGTGCATGTCGCTGTCAG	TGTCATCACTCTTAAGAGGCG
NUMA1 SE 72049419-72049539	TGGTCTCAAATTCCTTGGCT	CTCCAATGCGCCTTTTCAGG

SGO1 SE 20174249-20175055	CAGATAGACCAGTCACC AGG	GGTGGTGTAGTGGTAG GAGT
SGO1 SE 20175005-20175055	CTGAACTTCCAGGACAA GGA	GGTGGTGTAGCTGAAT CAAA
TMP2 SE 35685064-35685139	CGAGAGCCGAGCCAGAC AG	TCCTCCTCTGAGGCCAT CA
TMP2 MXE 35685064-35685139 no 35684732-35684807	CGAGAGCCGAGCCAGAC AG	TGGTGGAATACTCCTCC TCTG
