# Supplementary figures and images for: An ADAR1-dependent RNA editing event in the cyclin-dependent kinase CDK13 promotes thyroid cancer hallmarks
Source: Mol Cancer. 2021 Sep 8;20:115. doi: 10.1186/s12943-021-01401-y (PMC8424981; doi:10.1186/s12943-021-01401-y)

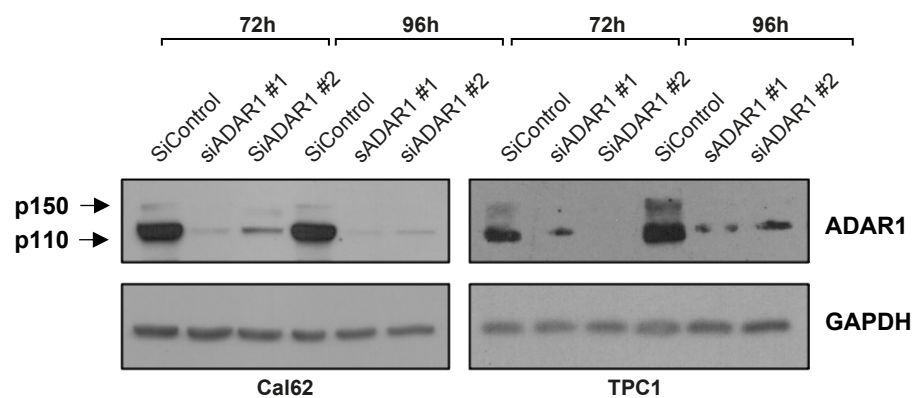

Supplement: Supplementary file 2 — Additional file 2: Supplementary Figure 1.ADAR1 silencing. Representative western blot of ADAR1 steady-state expression at the indicated time points after ADAR1 silencing in Cal62 and TPC1 cell lines. GAPDH was used as a loading control. [file 12943_2021_1401_MOESM2_ESM.pdf]

**A**

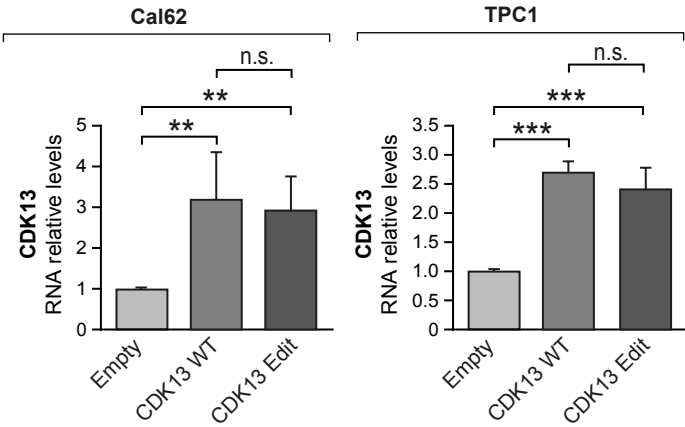

**B**

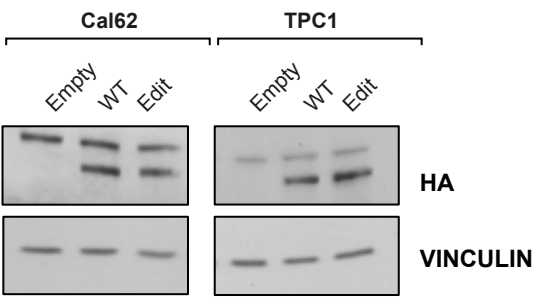

Supplement: Supplementary file 3 — Additional file 3: Supplementary Figure 2. CDK13 levels in cells stably expressing CDK13-WT, CDK13-Edit or the Empty vector. Cal62 and TPC1 were infected with lentivirus expressing the WT or the edited form of CDK13 (CDK13-WT and CDK13-Edit, respectively) or the corresponding empty vector, and selected using puromycin. (A) CDK13 levels in the indicated cell lines. (B) Representative western blotting for HA antibody in the indicated cell lines. Vinculin was used as loading control (n = 3). Error bars indicate standard deviations. Asterisks denote statistical significance compared with siControl treatment assessed by Student’s t-test (two-tailed). n.s: non significative p > 0.05, ** p < 0.01, *** p < 0.001. [file 12943_2021_1401_MOESM3_ESM.pdf]

**A**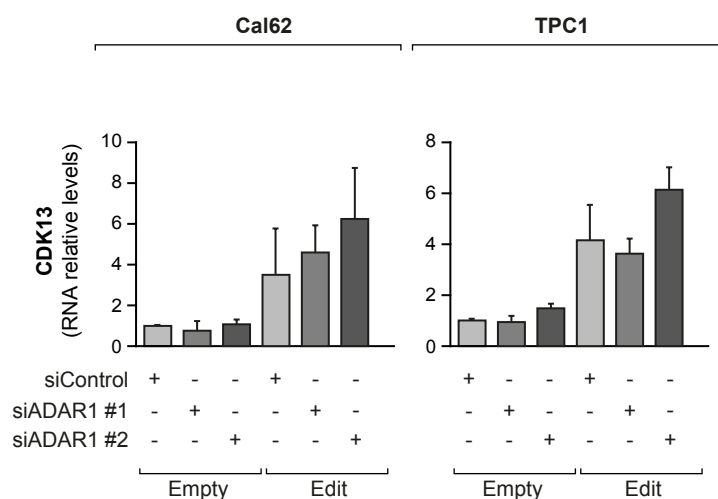**B**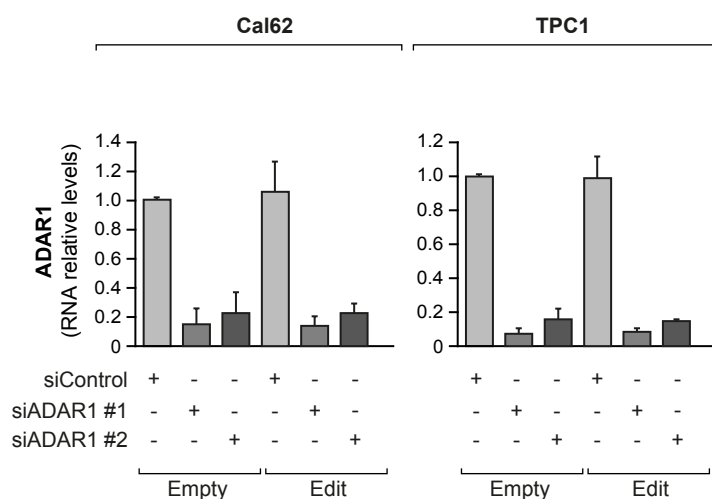**C**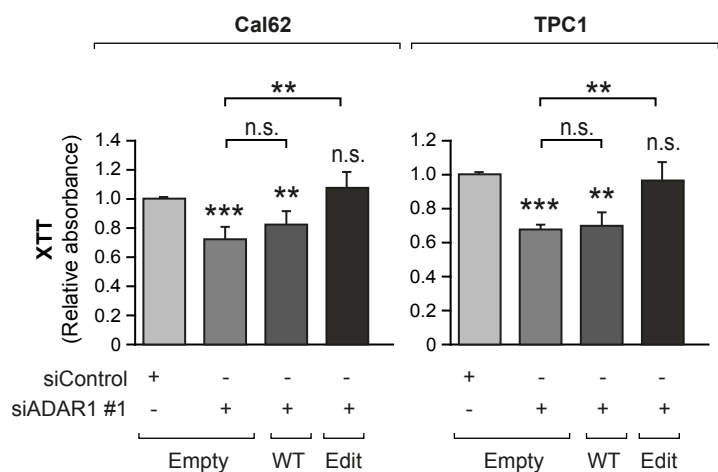

Supplement: Supplementary file 4 — Additional file 4: Supplementary Figure 3. CDK13 overexpression and ADAR1 silencing in Cal62 and TPC1 cell lines. RNA relative levels for CDK13 (A) and ADAR1 (B) in Cal62 and TPC1 cell lines stably expressing CDK13-Edit 72 hours after ADAR1 siRNA (siADAR1 #1 and #2) or Control siRNA (siControl) transfection. (C) Stably-transduced Cal62 and TPC1 cells with CDK13-Edit, CDK13-WT or empty vector were silenced for ADAR1 and were assayed for cell viability by XTT dye reduction (n = 4). Asterisks denote statistical significance assessed by Student’s t-test (two-tailed). n.s: non significative p > 0.05, ** p < 0.01, *** p < 0.001. [file 12943_2021_1401_MOESM4_ESM.pdf]
