# Supplementary material for: An ADAR1-dependent RNA editing event in the cyclin-dependent kinase CDK13 promotes thyroid cancer hallmarks
Source: Mol Cancer. 2021 Sep 8;20:115. doi: 10.1186/s12943-021-01401-y (PMC8424981; doi:10.1186/s12943-021-01401-y)

A

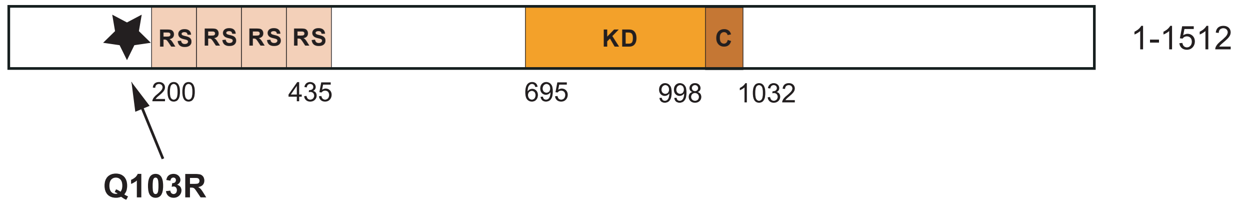

B

WT Predicted bipartite NLS		
**Pos.**	**Sequence**	**Score**
88	EVKRLARGKRRAGGR**Q**KRRRGP	8.6
91	RLARGKRRAGGR**Q**KRRRGPRAGQEAEKRRVF	5
97	RRAGGR**Q**KRRRGPRAGQEAEKRRVFS	5.5
676	SKRRPKICGPRYGETKEKDIDWGKRCVDK	5.3
999	RDVEPSKMPPPDLPLWQDCHELWSKKRRRQK	5.9

Edit Predicted bipartite NLS		
**Pos.**	**Sequence**	**Score**
88	EVKRLARGKRRAGGR**R**KRRRGP	13.6
97	RRAGGR**R**KRRRGPRAGQEAEKRRVFS	8
97	RRAGGR**R**KRRRGPRAGQEAEKRRVF	6
97	RRAGGR**R**KRRRGPRAGQEAEKRRVFSLP	5.2
676	SKRRPKICGPRYGETKEKDIDWGKRCVDK	5.3
999	RDVEPSKMPPPDLPLWQDCHELWSKKRRRQK	5.9

C

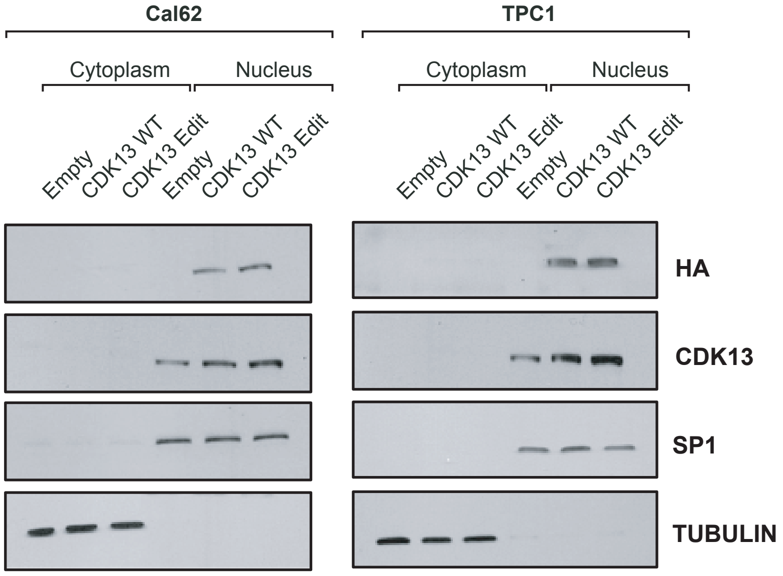

Supplement: Supplementary file 5 — Additional file 5: Supplementary Figure 4. Both CDK13-WT and CDK13-Edit are located in the cell nucleus. (A) Schematic representation of the CDK13 domain structure and the CDK13 editing event. Numbers below indicate the amino acid position. Abbreviations: RS: arginine/serine-rich; KD: kinase domain; C: C-terminal extension. (B) Predicted bipartite NLS (Nuclear Localization Sequence) in the WT and c.308 A>G edited form of CDK13. Predictions were performed using NLS mapper (http://nls-mapper.iab.keio.ac.jp). (C) Representative western blotting for HA and CDK13 in the indicated Cal62 and TPC1 cells after cytoplasm-nucleus fractionation. SP1 and tubulin were used as loading control for the nucleus and the cytoplasm, respectively. [file 12943_2021_1401_MOESM5_ESM.pdf]
